# A Comparative Study of Baseline Heart Rate Variability, Sleep Quality, and Oxidative Stress Levels in Hypertensive Versus Normotensive Subjects: A Cross-Sectional Study

**DOI:** 10.7759/cureus.25855

**Published:** 2022-06-11

**Authors:** Prabin Kharibam, Monika Pathania, Manisha Naithani, Yogesh Singh, Yogesh Bahurupi, Minakshi Dhar, Shashi R Yadav, Nitesh Singh

**Affiliations:** 1 Internal Medicine, All India Institute of Medical Sciences, Rishikesh, Rishikesh, IND; 2 Biochemistry, All India Institute of Medical Sciences, Rishikesh, Rishikesh, IND; 3 Physiology, All India Institute of Medical Sciences, Rishikesh, Rishikesh, IND; 4 Community and Family Medicine, All India Institute of Medical Sciences, Rishikesh, Rishikesh, IND

**Keywords:** hypertension, sleep quality, malondialdehyde, oxidative stress, heart rate variability

## Abstract

Objectives: To understand sleep quality, oxidative stress levels, and heart rate variability (HRV) in subjects with hypertension. This study aims to create baseline data in hypertensive subjects to research the possibility of further estimating the risk of developing cardiovascular mortality and morbidity in a patient with hypertension.

Design and methods: This analytical cross-sectional study, encompassing 128 study subjects of both genders, with 64 hypertensive subjects, analyse the co-relation of sleep quality, malondialdehyde, and heart rate variability in hypertensive and normotensive subjects. The study was done in a tertiary teaching institute in northern India for 14 months. Descriptive statistics were used, and the independent t-test, Mann-Whitney U test, and Chi-square were used to find the association among the variables. Linear regression was used to estimate the effect of blood pressure on malondialdehyde levels.

Results: Subjects with hypertension were found to have poor sleep quality (Global PSQI score ≥5, p=0.0001) and an increased malondialdehyde level (0.30303±0.17193 µM/L, p=0.0001). The hypertensive subjects were found to have lower parasympathetic activity as indicated by low high frequency (2.79463±473.220280; p=0.0001) and increased sympathetic activity; low frequency/high frequency (2.29823±2.792441; p=0.0001). Multivariate linear regression predicts that with one unit increase in systolic blood pressure, the malondialdehyde level increases by 0.006 units (p=0.002; 95% CI).

Conclusion: Among the hypertensive group, there is significantly increased oxidative stress level, poor quality of sleep, and increased sympathetic activity, thereby predisposing the subjects to increased risk of cardiac morbidity and mortality.

## Introduction

Hypertension continuously affects adults worldwide, with an estimated prevalence of 1.28 billion adults aged 30-79 years. Two-thirds of these hypertensive patients belong to low- and middle-income countries [1]. It has been observed that the overall prevalence of hypertension has been steadily increasing in India, with a high rate of variability in rural and urban areas. Although a lower prevalence of hypertension was noted earlier in the rural population than in the urban population, there is an increasing prevalence of hypertension in the rural population with changing lifestyles and urbanization [2]. Currently, hypertension accounts for 13.5% of the total mortality worldwide [3]. Hypertension remains asymptomatic and is usually identified during a routine checkup or when a complication sets in. It is accompanied by an increase in the renal and cardiovascular rates of morbidity and mortality, leading to premature death. It also warrants a prediction of the early reliability of cardiac morbidity and mortality [4].

The autonomic nervous system (ANS) acts as the primary regulator to control the sinus rhythm's heart rate (HR). The average HR at the time of resting is linked to the sympathovagal balance of the ANS, which is why further assessment provides attribution to the effect of the vagal and sympathetic input inside the sinoatrial (SA) node [5]. Heart rate variability (HRV) has been considered a noninvasive tool for diagnosing the overall integrity of the ANS. HRV is vital for identifying all subjects at a higher risk of developing hypertension. The integrity of the autonomic modulation of the HR is evaluated using linear and nonlinear analyses of HRV. The main positive feature of HRV is that it is cost-effective and noninvasive, which also helps generate an excellent diagnostic modality. The HRV method has been found to be very helpful, especially in developing countries where deaths and illnesses caused by heart problems are rising quickly [6].

Insomnia and disturbed sleep are usually linked together to increase the prevalence and incidence of hypertension [7]. HR and blood pressure (BP) remain altered during sleep, which the ANS primarily automates. The sleep cycle consists of rapid eye movement (REM) and non-rapid eye movement (NREM). NREM accounts for 75-80% of sleep duration [8]. An increase in parasympathetic activity during NREM sleep reduces HR, leading to decreased cardiac output and a reduced peripheral vasomotor tone, resulting in reduced BP. In contrast, REM is linked with sympathetic activity, which increases blood pressure. Individuals who experience disturbed sleep patterns are more in REM sleep, leading to non-dipping of blood pressure, which is associated with slow coronary flow and leads to cardiac mortality and morbidity [8]. A self-rated questionnaire, the Pittsburgh Sleep Quality Index (PSQI), helps assess the quality of sleep of an individual. PSQI helps evaluate the pattern of disturbed sleep, which can be correlated with hypertensive individuals [9]. The HRV decreases in subjects with poor sleep and hypertension and increases the risk of cardiac mortality [10].

Generally, patients with hypertension tend to develop several conditions that further accelerate the atherogenic process, such as platelet aggregation, platelet and leukocyte adhesion to the endothelium, and an increased number of free radicals [11]. The end products of lipid peroxidation are malondialdehyde (MDA) and free radicals. The formation of free radicals deactivates nitrogen oxide (NO), and decreased NO in the vascular endothelium causes a defect in endothelial vasodilation [12]. Increased free radicals, decreased NO levels, and a change in arachidonic acid metabolism lead to an accelerated process of atherosclerosis in the blood vessels [13]. The malondialdehyde level indirectly measures the oxidative stress levels in our body, with its increased level indicating an increased free radical and ultimately increased atherogenesis, predisposing the individual to cardiac mortality and morbidity.

This study aims to provide baseline data for future researchers to create a foundation for reducing the risks and help in the early detection of hypertension using a cost-effective, noninvasive tool, HRV, and further reaffirm our knowledge of the relationship between poor sleep and hypertension. The study aimed to compare the oxidative stress level among hypertensive and normotensive individuals and explore future modalities to prevent or treat hypertension with the baseline data collected. The study will also explore the relationship between the HRV, oxidative stress level, and poor sleep in a hypertensive individual.

## Materials and methods

All patients provided written informed consent before any study procedure in this analytical cross-sectional study. The study was compliant with ethical principles aligned with the Declaration of Helsinki, Good Clinical Practice guidelines, and local laws and regulations. The study was conducted in the LifeStyle disease clinic OPD, a special clinic under the Department of Internal Medicine, All India Institute of Medical Sciences, Rishikesh, for 14 months, from April 2020 to October 2021.

Included subjects were hypertensive and normotensive subjects aged between 25 and 45 years and willing to give written informed consent and consent to adhere to the study-specified procedures. Patients were excluded if they had comorbidities (diabetes mellitus/cerebrovascular accident/chronic obstructive pulmonary disease/chronic kidney disease), secondary causes of hypertension, a negative pregnancy urine test, a history of major psychiatric disorder or cognitive impairment, current dietary supplements (vitamin E or vitamin C) or medications affecting the autonomic nervous system, were obese (>25 kg/m^2^), or had any diagnosed sleep disorder (obstructive sleep apnea syndrome, narcolepsy, restless leg syndrome), working on a night shift, long-term use of immunosuppressants (e.g., steroids, chemotherapy, tumor necrosis factor (TNF)-inhibitors) and use of highly active antiretroviral therapy (HAART) for human immunodeficiency virus (HIV) patients. After reviewing related literature and using G*power (version-3.1.9.3), keeping alpha error = 0.05, equal sample size from two proportions (r = 1), the effect size is 0.05. Then the required sample size for the two groups to achieve an 80% power (β = 0.80), 64 subjects will be recruited in each group totaling 128 subjects with a confidence level of 95%. As per the standard protocol, BP was measured according to the American College of Cardiology and American Heart Association (ACC/AHA) 2017 guidelines. BP was defined as systolic blood pressure of >130 or diastolic blood pressure of >80 on an average of ≥2 readings on ≥2 occasions or patients already on antihypertensive treatment.

**Figure 1:**
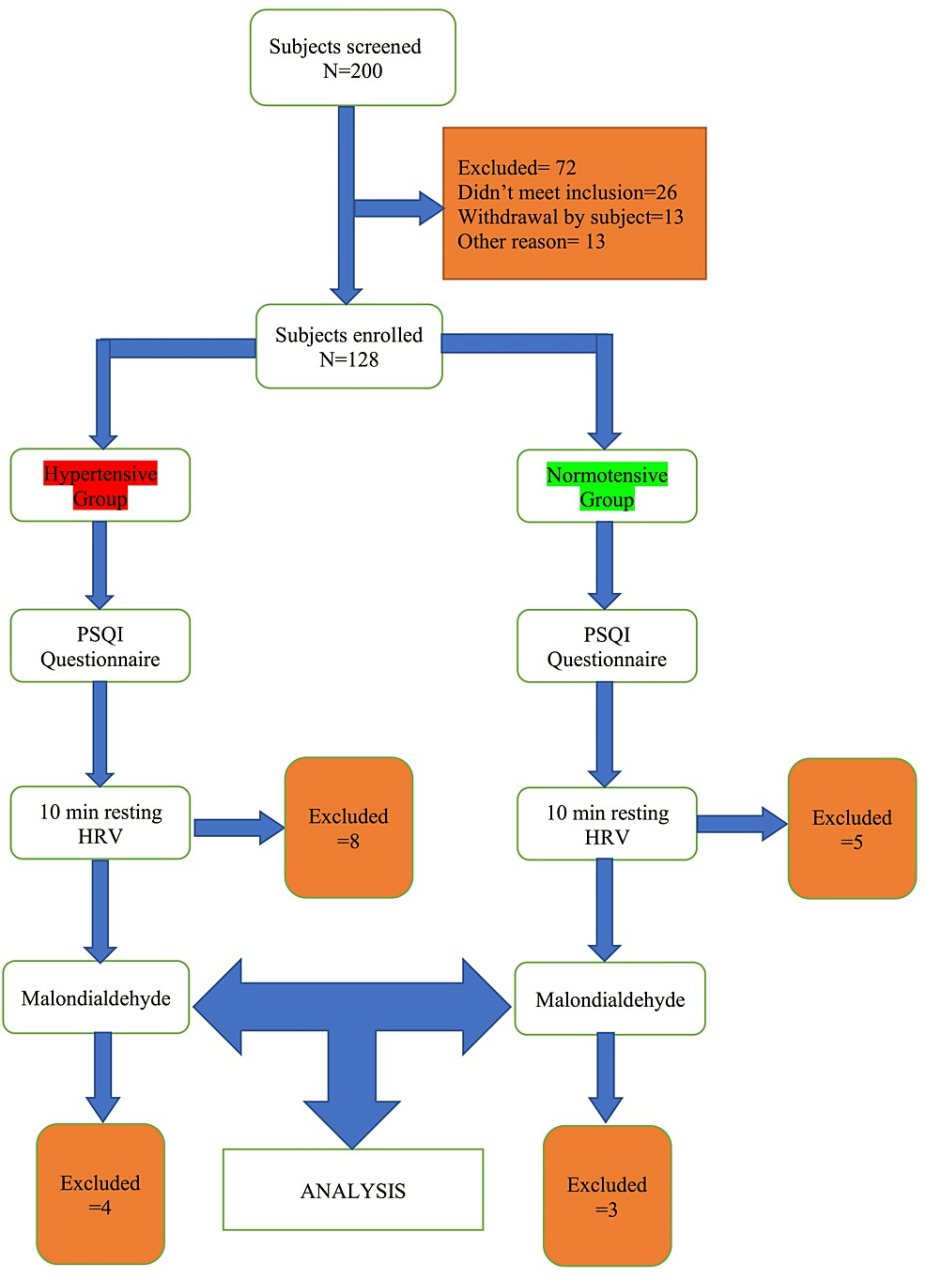
Flow diagram.

Two hundred subjects were screened, of which 72 were excluded from the study. Twenty-six did not meet inclusion criteria, and 13 withdrew from the study before enrolling. Thirteen subjects were excluded from the study due to nonadherence to protocol. Included subjects were enrolled in either group, and baseline data were entered into a case proforma following the protocol described in Figure 1.

A self-reported questionnaire, PSQI, was read out or given to them, and its data were saved along with the case record form. The individual's sleep quality is measured for one month using the PSQI. The PSQI questionnaire covers seven components and includes 19 individual items. These components consist of sleep latency, quality of subjective sleep, sleep disturbances, duration of sleep, the efficiency of habitual sleep, daytime dysfunction, and use of sleep as a medication. The patient fitting the criteria was called the next day with advice to refrain from eating, drinking alcoholic or caffeinated beverages, and smoking for at least 12 hours before the examination. Subjects were asked to rest in a supine position for at least 10 minutes, after which a resting ECG with lead II was taken for 10 minutes. Pre-processing of the HRV signal (tachogram) was done according to the guidelines provided by the Task Force (by the Board of the European Society of Cardiology, 1995). The HRV result was analysed using LabChart Pro v8.1.17 (ADInstruments, São Paulo, Brazil) with beat classification, keeping an RR interval of 400-1, 600 ms, and complexity of 1-1.5.

**Figure 2:**
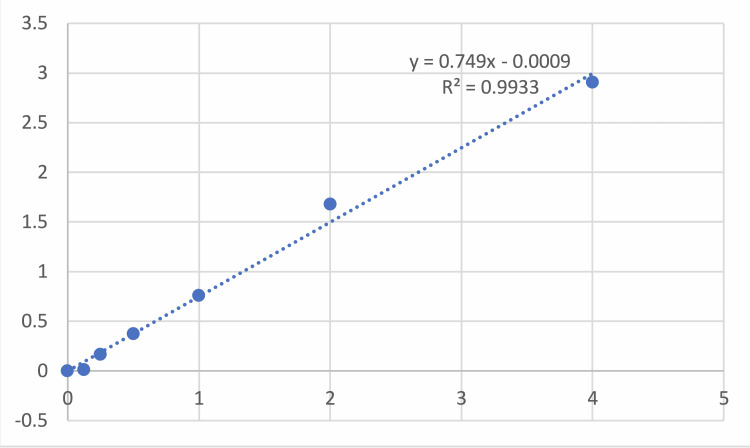
Standard graph to measure the unknown concentration of malondialdehyde.

Venous blood was drawn from the participating subjects, taking all aseptic precautions following HRV in a plain red vial. The serum was separated after centrifugation and was stored at −800 °C till further procedure. Malondialdehyde was done using the colourimetric method. The malondialdehyde present in the specimen reacts with thiobarbituric acid in sodium sulphate to form a pink-coloured complex. The intensity of the coloured complex directly depends on the concentration of MDA levels present in the serum sample. The coloured complex developed was later extracted in n-butanol, which was finally read for the absorbance readings at 530 nm (Green filter) using a colorimeter filter or ELISA reader. The method was standardised using various concentrations of standard solution (Table 1) and a standard curve was plotted on the graph paper for measuring the reading of test results and expressed in micromoles/litre (µM/L) (Figure 2).

**Table 1:** Concentrations of the standard prepared. MDA: malondialdehyde; DDW: double distilled water; µM/L: micromole per liter; µM: micromole; µL: microliter.

Standards	Concentration of MDA (µM/L)	Quantity of 10µM Working stock (uL)	Volume of DDW (µL)	Final volume of standard solution (µL)
S1	0.125	3.125	246.88	250
S2	0.25	6.25	243.75	250
S3	0.5	12.5	237.5	250
S4	1	25	225	250
S5	2	50	200	250
S6	4	100	150	250
S7	8	200	50	250

The HRV and malondialdehyde levels were measured and analysed independently on the same day without revealing the subjects to the principal investigator (PI). Subjects with missing/incomplete data were omitted from the study. Age, sex, and BMI were matched at the baseline to decrease confounding factors between the two groups. All statistical analyses were performed using the IBM Statistical Package for Social Sciences, version 26.0 (SPSS, IBM Corp., Armonk, NY). The normality of the data was tested using the Kolmogorov-Smirnov test and the Shapiro-Wilk test. The data were then represented as mean, standard deviation (SD), and descriptive statistics, followed by the application of parametric and non-parametric tests based on normality results. Independent-sample t-tests were used to compare the mean scores between the two groups. The Mann-Whitney U test was used for blood pressure as it was not normally distributed. Chi-square was used to find the association between the categorical variables. A p-value of <0.05 was considered to be statistically significant.

## Results

Twenty-three subjects were excluded due to incomplete/missing data. As indicated in Table 2, baseline anthropometric characteristics like age, gender, and BMI of both groups were comparable. Alcohol, smoking, and diets were also comparable.

**Table 2:** Baseline anthropometric and demographic profile of the participated subjects of both groups. BMI: body mass index.

Variables	Categories		Hypertensive group (n=64) [n (%)/mean, SD]	Normotensive group (n=64) [n (%)/mean, SD]	p-value
Gender		Male	31(48.4%)	33(51.6%)	0.724
		Female	33(51.6%)	31(48.4%)	
Age (years)			36.59±6.171	35.34±6.032	0.249
BMI (kg/m^2^)			24.006±1.3421	23.568±1.586	0.166
Diet		Non-vegetarian	21(48.8%)	22(51.2%)	0.852
		Vegetarian	43(50.6%)	42(49.4%)	
Smoking		Yes	12(52.2%)	11(47.8%)	0.818
		No	52(49.5%)	53(50.5%)	
Alcohol		Yes	14(46.75%)	16(53.3%)	0.676
		No	50(51%)	48(49%)	

The mean blood pressure reading of each group is summarised in Table 3.

**Table 3:** Mean blood pressure reading of hypertensive and control groups. BP: blood pressure; SBP: systolic blood pressure; DBP: diastolic blood pressure.

Variable		Hypertensive group (mean) n=64	Normotensive group (mean) n=64	p-value
BP 1^st ^(mmHg)	SBP	148±14	117±10	0.001
	DBP	86±12	72±6	0.001
BP 2^nd ^(mmHg)	SBP	144±10	118±9	0.001
	DBP	84±9	71±6	0.001

It was observed that the global PSQI score was ≥5 (5.78±1.362) among the hypertensive group, p=0.0001. The hypertensive group had a higher malondialdehyde level than the normotensive group; 0.0303±0.171 µM/L vs 0.21±0.081 µM/L; p=0.0001. Patients with hypertension have a higher average heart rate, indicating a more sympathetic tone of the vasovagal system. It is also observed that the average RR interval is decreased among hypertensive subjects. Spectral analysis of the HRV shows us that the vagal activity is depressed among the hypertensive subjects, as indicated by the low HF (2.794±473.22), p=0.0001. The LF/HF ratio is higher among the hypertensive group, indicating increased sympathetic activity (Table 4).

**Table 4:** PSQI, malondialdehyde, and heart rate variability comparison among hypertensive and control group. PSQI: Pittsburgh Sleep Quality Index; RR: R-R interval; SDRR: standard deviation of RR interval; CVRR: co-efficient of variation of R-R interval; SD: standard deviation; SDSD: standard deviation of the differences between successive RR intervals; pRR50: percentage of adjacent RR intervals that differ from each other by >50 ms; VLFL: very low frequency; LF: low frequency; HF: high frequency; µM/L: micromole per litre; ms: millisecond; bpm: beats per minute.

Heart rate variability	Hypertensive group (n=64) [mean, SD]	Normotensive group (n=64) [mean, SD]	p-value
PSQI	5.78±1.362	4.28±1.638	0.0001
Malondialdehyde (µM/L)	0.303±0.171	0.210±0.0814	0.0001
Average RR (ms)	741.264±124.139	749.435±244.467	0.013
Median RR (ms)	738.78±125.114	748.60±243.988	0.016
SDRR (ms)	35.94±34.386	50.954±24.0822	0.532
CVRR	.047±.045	0.06±.0341	0.508
Average rate (bpm)	83.750±15.73	71.235±10.307	0.010
SD Rate (bpm)	3.611±2.584	4.177±2.0008	0.383
SDSD (ms)	33.052±49.285	47.197±35.189	0.586
RMSSD (ms)	33.038±49.245	1.136±536.046	0.081
pRR50 (%)	5.837±10.694	3.948±153.892	0.066
Total power (ms^2^)	996.174±1245.463	2.567±1916.809	0.001
VLF (ms^2^)	4.269±602.654	9.954±805.564	0.010
LF (ms^2^)	2.744±360.005	6.166±569.513	0.005
HF (ms^2^)	2.794±473.22	9.271±967.071	0.0001
LF/HF	2.298±2.792	1.086±1.254	0.0001
SD1 (ms)	37.527±115.73	33.372±24.876	0.065
SD2 (ms)	43.669±35.817	62.713±26.253	0.592

Multivariate linear regression of the model predicts the change in malondialdehyde level with an R square of 0.802 and an adjusted R square value of 0.584. One unit increase in SBP will increase malondialdehyde levels by 0.006 units (p-value = 0.002) with a 95% confidence interval between −0.005 and 0.003 (Tables 5-6).

**Table 5:** Multivariate linear regression model summary. ^a^Predictors: (constant), average rate (bpm), DBP 1st, BMI (kg/m^2^), age, SBP 2nd, DBP 2nd, SBP 1st, median RR (ms), average RR (ms).

R	R Square	Adjusted R square	Standard error of the estimate
0.802^a^	0.643	0.584	0.1108

**Table 6:** Multivariate linear regression: coefficients. BMI: body mass index; SBP: systolic blood pressure; DBP: diastolic blood pressure; RR: R-R interval; ms: millisecond; bpm: beats per minute.

Model	Unstandardised coefficients		Standardised coefficients			95% confidence interval	
	B	Standard error	Beta	t	p-value	Lower bound	Upper bound
(Constant)	−2.028	0.761		−2.664	0.010	−3.554	−0.502
Age	0.000	0.003	−0.014	−0.139	0.890	−0.006	0.005
BMI	0.016	0.011	0.125	1.487	0.143	−0.006	0.038
SBP 1^st^	0.006	0.002	0.526	3.247	0.002	0.002	0.010
DBP 1^st^	0.000	0.002	−0.065	−0.472	0.639	−0.005	0.003
SBP 2^nd^	0.004	0.002	0.244	1.762	0.084	0.000	0.009
DBP 2^nd^	0.001	0.002	0.042	0.343	0.733	−0.004	0.005
Average RR (ms)	0.000	0.001	−0.143	−0.151	0.881	−0.003	0.002
Median RR (ms)	0.001	0.001	0.494	0.560	0.578	−0.002	0.003
Average rate (bpm)	0.001	0.004	0.109	0.292	0.771	−0.007	0.009

## Discussion

Our study recruited 128 study subjects, of whom 64 were hypertensive and 64 were normotensive. All adult patients aged 25-45 years were included in this study, whereas those with comorbidities, secondary causes of hypertension, a history of major psychiatric disorder, and cognitive impairment were excluded.

According to the demographic distribution of the study subjects, the mean age, sex, and BMI were similar between the two study groups. Several authors have also reported that BMI is positively associated with SBP and DBP [14]. Our study reported that the mean BMI of hypertensive patients was slightly higher, although statistically insignificant (p=0.166) than that of normotensive patients. On the other hand, the findings by Ahmad et al. recorded a lower BMI among hypertensive patients than among normotensive patients [15]. In a cross-sectional study, Cassani et al. found a significant linear correlation between BP and waist circumference [16]. Another cross-sectional study conducted in rural Wardha by Deshmukh et al. showed that BMI and waist circumference were positively correlated with BP [17]. BMI was comparable between the two groups in our study, as we kept a specific BMI limit as our exclusion criteria.

MDA is an end product of lipid peroxidation resulting from oxidative stress between antioxidants and reactive oxygen species [18]. It is an indirect indicator of increased free radicals in the body. Free radicals, thus, inhibit vascular endothelium relaxation, thereby increasing BP [19]. In our study, the MDA levels among hypertensive subjects were significantly higher (0.30303±0.17193 μM/L) than in those with normal BP. Similar to our study, Armas-Padilla et al. performed a cross-sectional study that recruited 63 study subjects (21 normotensive and 42 hypertensive subjects) to evaluate the NO status by measuring the serum and urinary NO levels and serum MDA as a marker of oxidative stress in healthy normotensive subjects and untreated essential stage 2 hypertensive patients [12]. The findings concluded that patients with essential hypertension, compared with normotensive individuals, had lower NO status, which may contribute to endothelial dysfunction in hypertension. Increased serum MDA levels in hypertensive individuals suggested an association between increased oxidative stress and higher BP. Ahmad et al. in their study reported that the MDA levels were significantly increased in stage I and II hypertensive subjects compared with those in normotensive individuals. They also observed a significant decrease in the MDA levels after six months of antihypertensive therapy in stage II hypertensive subjects than before the treatment (p<0.05). However, there was no significant difference in the MDA levels before and after treatment in stage I hypertensive subjects (p>0.05). This suggested that tight regulation of BP led to a decrease in oxidative stress among hypertensive subjects. Moreover, animal studies have supported the hypothesis that increased BP is associated with increased oxidative stress. Other studies by Li et al., Chaves et al., and Parslow et al. also pointed out an increase in serum MDA levels and decreased urinary NO levels among hypertensive subjects. In their follow-up, tight regulation of BP led to a decrease in the MDA level [18-20]. In a similar study, Tandon et al. observed that the serum MDA levels, 0.33±0.07 μM/L, among hypertensive subjects were significantly higher than those in normotensive subjects. They also observed that the levels of oxidative stress markers significantly decreased after three months of follow-up after treating hypertension [21].

In their study, Koichubekov et al. recruited 56 study subjects (32 cases and 24 controls) between the ages of 45 and 55 years and studied HRV parameters. The findings indicate that in hypertension, a vegetative balance shift occurs toward the sympathetic division of the ANS while the linear and nonlinear structures of HRV change. There is a decrease in the variability of the heart rhythm owing to a decrease in the power of periodic HF oscillations while simultaneously increasing the LF component of the spectrum. Among the hypertensive group, the present study showed a significant increase in variables such as average rate (bpm), total power (ms^2^), and LF/HF ratio and a decrease in average RR interval (ms) and HF (ms^2^), indicating increased sympathetic tone of the ANS. Koichubekov et al., with relevance to our finding, reported in their study that HRV spectral components were higher in the hypertensive group than in the normal group [6]. Pal et al. also assessed the sympathovagal balance with HRV among pre-hypertensive and hypertensive individuals and concluded that there is significant vagal inhibition among hypertensive subjects, leading to prominent sympathetic activity [22].

This study showed a significant association between sleep quality and the study group. The majority of patients (77%) with hypertension had poor sleep quality. However, 23% of the total study subjects had poor sleep quality in the control group participants. Moreover, the global PSQI score increased significantly among hypertensive subjects (score >5). Bruno et al. found that 38.2% of Italian patients with hypertension had poor sleep quality [23]. Kara and Tenekeci et al. reported a poor sleep quality prevalence of 42.4% in Nigerian patients with hypertension [24]. A Turkish study reported that 17 (43.6%) of 39 patients with stage 1 hypertension had poor sleep quality at baseline. These studies defined a global PSQI score >5 as poor sleep quality. Our findings also showed that female patients were more likely to have poor sleep quality than male patients. Our findings were similar to those of the study conducted by Huang et al. [25]. In contrast, other studies have not detected sex differences in sleep quality in patients with hypertension in Nigeria [26] and China, as well as older adults in Taiwan (aged ≥60 years) [27]. Moreover, a study conducted by Sajjadieh et al. reported that poor sleep quality is adversely associated with HRV, HR, and BP [10]. Reduced HRV is also associated with poor outcomes in patients with post-myocardial infarction [28].

## Conclusions

This study attempted to assess the baseline levels of heart rate variability, malondialdehyde levels, and sleep quality among hypertensive and normotensive individuals and their correlations. Our study showed that the participants' sleep quality was significantly impaired in hypertensive individuals. Moreover, the mean malondialdehyde level was higher in the hypertensive group than in the normotensive group. Individuals with decreased heart rate variability should be closely monitored for blood pressure, as they have a high propensity to develop hypertension owing to increased sympathetic activity. Physical activity is suggested to help prevent and eliminate sleep problems because of its low cost and fewer side effects. Moreover, further research is required to study the possible association between poor sleep quality and variation in heart rate. It is essential to determine how proteins are differentially oxidised and activated to fully understand the functional impact of oxidative stress on health and disease and possible intervention with antioxidants. The study will be used as baseline data for a further longitudinal study with a large sample size to explore the hypothesis of reducing cardiac mortality and morbidity by maintaining good sleep hygiene and the role of antioxidants in reducing hypertension. Furthermore, HRV needs to be incorporated into managing hypertension to stratify the risk.

## Figures and Tables

**Figure 3:**
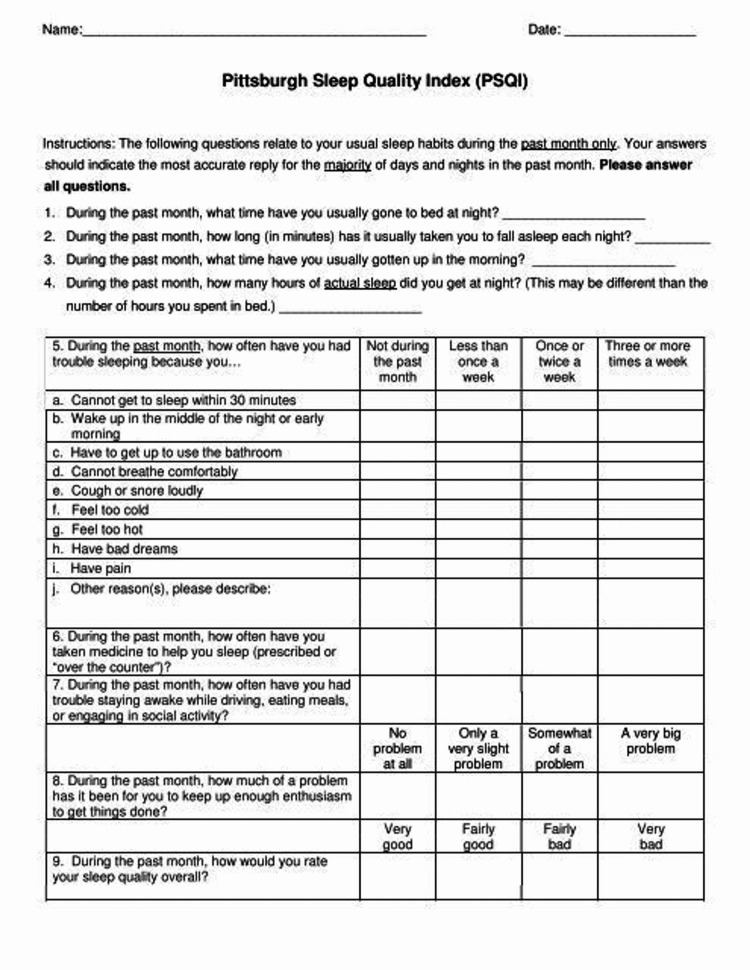
PSQI questionnaire

## Appendices

Malondialdehyde estimation

Sample Collection

Three millilitres of venous blood was drawn from each subject in a sterilised manner from the median cubital vein in a plain red vial (BD, USA). The whole blood was allowed to clot for 10 minutes before being centrifuged at 3000 rpm for five minutes to separate the serum, which was then collected in a sterile Eppendorf microcentrifuge tube (Eppendorf, Germany) and stored at −800 °C until the next procedure.

MDA Analysis

Instruments Used:

20 ml Graduated tubes

Ice filled Container

Centrifuge

Boiling Water Bath

Vortex

ELISA Plate

ELISA Reader

Reagents Used and Their Preparation

MDA: MDA is required to prepare different Standard concentrations based on which samples are quantified.

TCA: 20% TCA was prepared by weighing 20 g of pure solute Trichloroacetic acid in 100ml of DDW.

H_2_SO_4_: 0.05 M sulphuric acid was prepared by adding 2.8 mL of Concentrated Sulphuric acid in 1 litre of DDW.

Sodium sulphate solution: 2 M of sodium sulphate solution was prepared by dissolving 28.4 g of anhydrous sodium sulphate in 90 mL of DDW with constant heating and stirring. Further final volume is made to 100 mL with DDW.

TBA: 0.67% of thiobarbituric acid was prepared by dissolving 670 mg of TBA in 100 mL of 2 M sodium sulphate solution.

n-Butanol/n-butyl alcohol: Readily and commercially available n-Butyl alcohol was used.

Sterile/double distilled water

Standard Preparation

MDA Standard solution was prepared from 500 µM stock solution purchased (EZAssay TBARS Estimation kit, Himedia, India).

Working stock standard for MDA: Prepared by diluting 0.2 mL of stock solution in 10 mL of 0.05 M H_2_SO_4_ to obtain 10 µM working stock solution.

Different concentrations of standards were prepared by diluting working stock according to the following table:

Principle of MDA

Lipoproteins were precipitated from the serum sample by adding 20% TCA. The malondialdehyde present in the specimen reacts with thiobarbituric acid in sodium sulphate to form a pink-coloured complex. The intensity of the coloured complex directly depends on the concentration of MDA levels present in the serum sample.

The coloured complex developed was later extracted in n-butanol, which was finally read for the absorbance readings at 530 nm (green filter) using a colorimeter filter or ELISA reader. The method was standardised using various concentrations of standard solution, and a standard curve was plotted on graph paper for measuring the reading of test results.

Procedure of MDA Estimation

Three clean, dry, and labelled test tubes were arranged in the test tube rack, and the following were pipetted out carefully in respective tubes as in Table 7

**Table 7:** Detailed procedure for malondialdehyde estimation.

REAGENTS	TEST (µl)	STANDARD (µl)	BLANK (µl)
Serum	250	-	-
Standard (10μM/L)	-	250	-
Double Distilled Water	-	-	250
20 % TCA	1250	1250	1250
Tubes were left to stand for 10 minutes at Room Temp			
Tubes were then centrifuged at 3500 rpm for 10 mins			
Supernatant was decanted, and the precipitate was processed with the following reagents.			
0.05M H_2_SO_4_	1250	1250	1250
0.67 % TBA	1500	1500	1500
All tubes were heated in a boiling water bath at 90^o^C for 30 minutes.			
Tubes were then cooled on ice.			
n-Butanol	2 ml	2 ml	2 ml
With vigorous shaking, resulted chromogen were obtained.			
Separation of organic phase by centrifugation at 3000rpm for 10 mins was done			
The organic phase was filtered and 200 µl pipetted respectively in different wells of ELISA plate.			

The ELISA plate wells were mixed well and read at 530 nm on the ELISA reader.

Patient case proforma

Participant no:                                                 Name:                                                                 

Age/Sex:                                                          Religion

History reported by:                                         Address:                                                            

Phone No:                                                         Occupation

Presenting complains: (with duration in chronological order) 

HOPI:

Past history: (with details of treatment and outcome status)

Treatment history: (including details of outside hospital treatments)

Family and close contacts history: 

Personal history: 

1.    Education-

2.    Married/unmarried -

3.    Occupation (monthly income, if any) - 

4.    Habits (details of smoking/alcohol) - 

5.    Details of hygiene habits (e.g., hand washing, disposal of sewage, etc.) - 

6.    Menstrual history (if female) - 

7.    Sexual history - 

Any STDs - (details including cure)

8.    Sleep pattern and mental status - 

9.    A dietary pattern including recent detailed food history up to 2 days before symptom onset- 

10.  Vaccinations status - 

11.  Travel history - 

Social history: (including socioeconomic status, surrounding lifestyles, home and habitus, occupation)

Physical examination: (with consent and hand hygiene)

1.     Mental status - 

2.    General appearance -

3.    Vitals: PR -                , RR -             , BP -                          , Temp -       SpO_2_ -      

4.    Skin -                        Height:                   Weight:                              BMI:

Inspections

Palpations

Percussions

Auscultation

5.     Chest (respiratory system)

6.     Chest (cardiovascular)

7.     Abdomen

8.    Genito-urinary-Rectal exam  -

9.     Musculoskeletal exam -

10.   Neurological exam -

11.    Investigation planned (to be sent for all participants)- 

·       Complete blood count

·       Blood urea

·       Serum creatinine

·       Serum electrolytes (Na, K, Cl, Ca)

·       Chest X-ray

·       12 lead ECG

·       Urine analysis 

·       Plasma glucose
